# Perioperative fluids and complications after pancreatoduodenectomy within an enhanced recovery pathway

**DOI:** 10.1038/s41598-020-74907-y

**Published:** 2020-10-21

**Authors:** Jérôme Gilgien, Martin Hübner, Nermin Halkic, Nicolas Demartines, Didier Roulin

**Affiliations:** Department of Visceral Surgery, Lausanne University Hospital (CHUV), University of Lausanne (UNIL), 1011 Lausanne, Switzerland

**Keywords:** Surgical oncology, Pancreatic disease, Pancreatic cancer

## Abstract

Optimized fluid management is a key component of enhanced recovery (ERAS) pathways. Implementation is challenging for pancreatoduodenectomy (PD) and clear guidance is missing in the respective protocol. The aim of this retrospective study was to evaluate the influence of perioperative intravenous (IV) fluid administration on postoperative complications. 164 consecutive patients undergoing PD within ERAS between October 2012 and June 2017 were included. Perioperative IV fluid and morbidity (Clavien classification and comprehensive complication index (CCI)) were assessed. A threshold of more than 4400 ml IV fluid during the first 24 h could be identified to predict occurrence of complications (area under ROC curve 0.71), with a positive and negative predictive value of 93 and 23% respectively. More than 4400 ml intravenous fluids during the first 24 h was an independent predictor of overall postoperative complications (adjusted odds ratio 4.40, 95% CI 1.47–13.19; p value = 0.008). Patients receiving ≥ 4400 ml were associated with increased overall complications (94 vs 77%; p value < 0.001), especially pulmonary complications (31 vs 16%; p value = 0.037), as well as a higher median CCI (33.7 vs 26.2; p value 0.041). This threshold of 4400 ml intravenous fluid might be a useful indicator for the management following pancreatoduodenectomy.

## Introduction

The outcomes following pancreatoduodenectomy (PD) have improved, with a drastic fall of postoperative mortality from 15 to 20% in the 80s^1^ to less than 2% in high-volume centres^2^ nowadays. These interventions still have a high morbidity and patients may need a delay up to 6 month to reach their preoperative quality of life^3,4^. In an effort to improve those outcomes, the concept of enhanced recovery (ERAS) is increasingly recommended for pancreatic surgery. ERAS pathway aims to reduce the surgical stress in order to shorten patient’s recovery and works in pancreatic surgery^5,6^. One of the cornerstone of enhanced recovery is balanced fluid therapy with avoidance of fluid overload. Uncontrolled perioperative fluid administration has potential deleterious impact on postoperative outcome. While fluid overload may lead to interstitial edema, hypovolemia may result in renal dysfunction. For these reasons, implementation of an enhanced recovery protocol for PD is challenging^7^ and specific guidance on defining fluid balance for PD are required.

This study aimed to assess the potential impact of perioperative fluid administration on postoperative outcomes after PD within an ERAS protocol.

## Results

A total of 178 consecutive patients underwent pancreatoduodenectomy in our institution during the study period. As 14 patients refused the use of their data, 164 patients were included in the analysis. Patients demographics and surgical characteristics are detailed in Table 1. The final diagnosis was a primary adenocarcinoma in most of the cases (82%), with 11 patients among them who underwent neoadjuvant chemotherapy. All operations were open PD without pylorus preservation. The overall compliance to the ERAS pathway was 62%, with 4% of missing data. The mean compliance was 99% for preoperative items (100% no oral bowel preparation, 98% oral carbohydrate drinks, 95% avoidance of long-acting sedatives, 99% thromboprophylaxis, 100% antibioprophylaxis, 100% postoperative nausea and vomiting prophylaxis), 87% for intraoperative items (99% epidural when not contraindicated, 100% upper-body heating cover, 88% removal of nasogastric tube at end of surgery, 62% early drain removal), and 32% for postoperative items (2% termination of urinary drainage on Postoperative Day (POD) 3, 83% of stimulation of gut motility, 99% postoperative use of epidural if applicable, 12% < 3500 ml IV fluids on POD 0, 7% termination of IV fluids on POD 2, 25% patient weight on POD1, 4% mobilization on day of surgery, 42% mobilization more than 4 h on POD1, 25% mobilization more than 6 h on POD 2, 34% mobilization more than 6 h on POD3, 17% 30 day follow up).

**Table 1:** Patients preoperative and surgical characteristics.

	Overall (n = 164)
Age, years, mean (SD)	65 (12)
Gender (m:f)	86:78
BMI, kg/m^2^, mean (SD)	25.6 (4.7)
**ASA, n (%)**	
1–2	109 (66)
3–4	55 (34)
**Preoperative WHO performance status, n (%)**	
0	19 (12)
1–3	145 (88)
Active smoker, n (%)	44 (27)
Diabetes, n (%)	28 (17)
Malignant tumor, n (%)	142 (86)
Neoadjuvant chemotherapy, n (%)	11 (7)
Operation length, min, median (IQR),	335 (288–378)
Venous resection, n (%)	37 (22)
**Pancreatic texture, n (%)**	
Soft	80 (49)
Hard	65 (40)
Unknown	19 (11)
**Pancreatic main duct, n (%)**	
Dilated > 2 mm	71 (43)
Non-dilated	85 (52)
Unknown	8 (5)
Intraoperative infusion of vasoactive drugs, n (%)	163 (99)
**Fluid administration guidance, n (%)**	
Pulse pressure variation	114 (69)
Oesophageal doppler	11 (7)
Transoesophageal echocardiography	2 (1)
Unknown	37 (23)
Estimated blood loss, ml, median (IQR),	440 (300–800)

*ASA* American Society of Anesthesiology; *BMI* Body Mass Index, *SD* standard deviation, *n* number, *IQR* interquartile range; *WHO* World Health Organization; *IV* intravenous; *POD* postoperative Day.

### Perioperative fluid and overall complications

The median total amount of IV fluids POD 0 was 5005 ml (IQR 3963–6124). The median intraoperative IV fluid was 3500 ml (IQR 2575–4500) and was composed by crystalloids (median 3000 ml, IQR 2175–3500) and colloids (median 500 ml, IQR 0–1000). The median intraoperative infusion rate was 9 ml/kg/h (IQR 7–11). The amount of IV fluids POD 0 was composed by the addition of intraoperative, and postoperative IV fluids, and both were higher in patients with complications (3500 ml (IQR: 2712–4500) vs 3000 ml (IQR 2000–3687); p value 0.034, and 1400 ml (IQR 1100–1860) vs 1165 (IQR 770–1605); p value 0.024, respectively).

**Figure 1:**
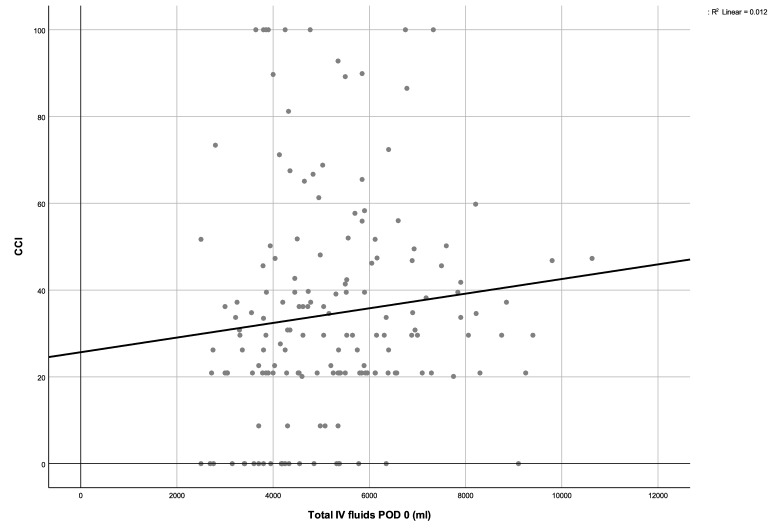
Correlation between intravenous (IV) fluids on postoperative day (POD) 0 fluids and Comprehensive Complication Index (CCI). Spearman’s rank correlation coefficient (Rho = 0.168, p value = 0.020) between the volume of intravenous fluid within the first postoperative day (IV fluids POD 0) and the Comprehensive Complication Index (CCI).

As postoperative complications were higher in patients with increased perioperative fluids, the potential correlation between perioperative fluids and postoperative complications was assessed. As presented in Fig. 1, the amount of IV fluids POD 0 weakly correlated with CCI (r = 0.168, p value = 0.020).

### Fluid threshold

**Figure 2:**
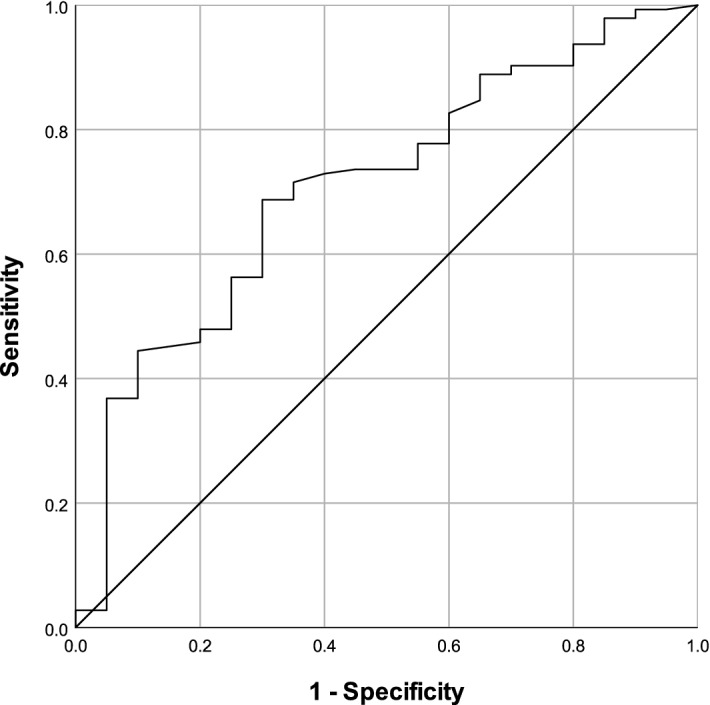
Receiver Operating Characteristic (ROC) curve of intravenous fluids on postoperative day 0 for overall complications. The area under the curve was 0.71 (95% CI 0.59–0.83). The optimal threshold point based on Youden index was 4400 ml.

In order to identify a critical threshold, a ROC curve analysis of IV fluids POD 0 (ml/24 h) and overall complications was performed (*Fig. **2**)*. The Area Under the Curve (AUC) was 0.71 (95% CI 0.59–0.83), which is above the 0.70 cut-off indicating moderate accuracy^8^. The optimal threshold of IV fluids POD 0 as predictor of complications was set at 4400 ml. This threshold provided a sensitivity of 69% and a specificity of 63% with a positive predictive value of 93% and a negative predictive value of 23%. The ROC curve analysis for IV fluids POD 0 and major complications was not contributive (AUC = 0.55). Further ROC curve analyses of IV fluids on POD taking account the body weight (ml/kg/24 h) found an AUC of 0.69 for overall complications and an AUC of 0.49 for major complications. When considering the infusion rate of intraoperative fluids (ml/kg/h) the AUC for overall and major complications were 0.58 and 0.42, respectively.

### Predictors for overall and major complications

In order to evaluate the potential predictive role of perioperative fluid on postoperative complications, a binary logistic regression analysis with preoperative and perioperative characteristics including the identified 4400 ml threshold of IV fluids on POD 0 was performed and presented in Tables 2 and 3. On multivariable analysis, the 4400 ml IV fluids POD 0 was an independent predictor of overall postoperative complications (adjusted OR 4.40, 95% CI 1.47–13.19; p value = 0.008), but not of major complications.

**Table 2:** Predictive factors for overall complications at 30 post-operative days.

	Univariable analysis			Multivariable analysis		
	OR	95% CI	p value	Adjusted OR	95% CI	p value
Age > 70 years	1.65	0.57–4.79	0.361			
Gender (female)	0.74	0.28–2.01	0.561			
BMI > 25 kg/m^2^	1.12	0.35–2.48	0.807			
ASA III/IV	0.93	0.19–1.7	0.882			
WHO performance > 0	2.15	0.64–7.28	0.218			
Active smoking	1.54	0.49–4.8)	0.465			
Diabetes	0.80	0.25–2.60	0.711			
Malignant tumor	0.69	0.15–3.20	0.633			
Neoadjuvant Chemotherapy	1.42	0.02–11.71	0.745			
Soft pancreatic texture	1.64	0.61–4.42	0.331			
Non-dilated pancreatic main duct	4.54	1.41–14.63	0.011	5.32	1.59–17.78	0.007
Length surgery > 300 min	1.54	0.60–3.96	0.372			
Intraoperative blood loss > 400 ml	1.18	0.45–3.08	0.738			
Venous resection	0.84	0.28–2.51	0.748			
IV POD 0 ≥ 4400 ml	4.82	1.74–13.33	0.002	4.40	1.47–13.19	0.008

*OR* odd ratio, *CI* confidence interval; BMI, body mass index; *ASA* American Society of Anesthesiologists; *WHO* World Health Organization; *IV* intravenous; *POD* post-operative day.

**Table 3:** Predictive factors for major complications at 30 post-operative days.

	Univariable analysis			Multivariable analysis		
	OR	95% CI	p value	Adjusted OR	95% CI	p value
Age > 70 years	1.41	0.73–2.70	0.303			
Gender (female)	0.35	0.18–0.66	0.001	0.39	0.19–0.79	0.009
BMI > 25 kg/m^2^	0.97	0.52–1.81	0.926			
ASA III/IV	1.85	0.96–3.57	0.066	1.92	0.94–3.93	0.075
WHO performance > 0	4.58	1.28–16.41	0.019	5.63	1.46–21.71	0.012
Active smoking	0.91	0.45–1.83	0.781			
Diabetes	0.70	0.30–1.64	0.41			
Malignant tumor	1.36	0.54–3.44	0.521			
Neoadjuvant chemotherapy	0.75	0.21–2.68	0.662			
Soft pancreatic texture	1.55	0.79–3.02	0.201			
Non-dilated pancreatic main duct	2.35	1.22–4.52	0.011	2.22	1.10–4.50	0.026
Length surgery > 300 min	1.14	0.60–2.17	0.697			
Intraoperative blood loss > 400 ml	1.21	0.64–2.29	0.554			
Venous resection	0.73	0.35–1.52	0.402			
IV POD 0 ≥ 4400 ml	1.24	0.65–2.37	0.506			

OR: Odd ratio, CI : Confidence Interval; BMI, body mass index ; ASA, American Society of Anesthesiologists; WHO, World Health Organization ; IV: intravenous; POD, post-operative day.

### Postoperative outcomes according to critical fluid threshold

According to this 4400 ml of IV fluids POD 0, postoperative outcome was compared as shown in Table 4. There were 61 patients who received less than 4400 ml and 103 patients who received 4400 ml or more. The overall complication rate and the CCI were significantly higher in the ≥ 4400 ml group (94 vs 77%; p value = 0.001, and 33.7 vs 26.2; p value = 0.041, respectively). The 90 days after surgery mortality was not different in both groups: 3% in the < 4400 ml group (one patient died of hemorrhagic shock and one patient of septic shock) and 2% in the ≥ 4400 ml group (one patient died of bleeding shock and one patient of disease progression with carcinosis after 61 postoperative days); p value = 0.629. The rate of postoperative pulmonary complications was significantly increased in patients receiving higher perioperative volume (31 vs 16%; p value = 0.037). The increased rate of respiratory complications in patients receiving more than 4400 ml was especially observed for minor (Clavien I-II) complications (21 vs 5%; p value = 0.006). There was no significant difference among detailed pulmonary complications, such as atelectasis (8 vs 5%; p value = 0.748), pneumonia (18 vs 10%; p value = 0.179), pleural effusion (12 vs 10%; p value = 0.800), respiratory failure (10 vs 5%; p value = 0.332), and pneumothorax (1 vs 0%; p value = 1.000). Regarding hemodynamic related complications, there was no significant difference in terms of cardio-vascular dysfunction (13 vs 10%; p value 0.500), and of renal failure (3 vs 5%; p value = 0.322). Between the two groups. In the < 4400 ml group, one patient had a renal failure classified AKIN (Acute Kidney Injury Network) 1 and one patient AKIN. 3. In the group with more than 4400 ml, two patients were classified AKIN 1 and three patients AKIN 3. No significant difference in pancreatic surgery specific complications such as delayed gastric emptying or pancreatic fistula was observed between both groups. The use of postoperative nasogastric tube was 3% (2/61) in the < 4400 ml group compared to 17% (18/103) (p value = 0.007). The rate of nasogastric tube reinsertion was comparable (43% vs 42%; p value = 0.913). The rate of postoperative parenteral nutrition started in the postoperative period was not different between both groups (55 vs 49%; p value = 1.000). The number of relapartomy needed for postoperative hemorrhage was 8 (8%) in patients with ≥ 4400 ml compared to 5 (8%) in patients with < 4400 ml (p value = 1.000). The proportion of patients requiring intensive care unit was 21% in the < 4400 ml group and 19% in the ≥ 4400 ml group (p value = 0.841).

**Table 4:** Postoperative outcomes stratified by perioperative intravenous fluids.

	IV fluids POD 0 < 4400 n = 61	IV fluids POD 0 ≥ 4400 n = 103	p-value
**Complications**			
Overall, n (%)	47 (77)	97 (94)	0.001
Major (IIIa–IVb), n (%)	24 (39)	46 (45)	0.506
Minor (I–II), n (%)	23 (38)	51 (50)	0.149
Mortality 90 days after surgery, n (%)	2 (3)	2 (2)	0.629
CCI, median (IQR)	26.2 (4.4–38.4)	33.7 (20.9–47.3)	0.041
Pulmonary complications, n (%)	10 (16)	32(31)	0.037
Infectious complications, n (%)	20 (33)	48 (47))	0.083
Cardio-vascular dysfunction, n (%)	8 (13)	10 (10)	0.500
Renal failure, n (%)	2 (3)	5 (5)	0.322
**Pancreas specific complications**			
Delayed gastric emptying, n (%)	31 (51)	49 (48)	0.810
Grade A, n (%)	15 (25)	29 (28)	0.716
Grade B, n (%)	8 (13)	13 (13)	1.000
Grade C, n (%)	8 (13)	7 (7)	0.261
Pancreatic fistula (Grade B-C), n (%)	11 (18)	25 (24)	0.436
Biliary fistula n (%)	3 (5)	6 (6)	1.000
Postoperative hemorrhage, n (%)	11 (18)	16 (16)	0.677
Grade A, n (%)	4 (6)	2 (2)	0.196
Grade B, n (%)	1 (2)	5 (5)	0.417
Grade C, n (%)	6 (10)	8 (8)	0.573
**Lengths of stay**			
Primary, median (IQR)	14 (11–23)	19 (14–27)	0.034
Intensive care unit, median (IQR)	0 (0–0)	0 (0–0)	0.779
Total, median (IQR)	15 (11–23)	19 (14–29)	0.015

*n* number, *CCI* Comprehensive Complication Index, *IQR* interquartile range, *ml* milliliter.

## Discussion

This study described a higher administration of perioperative IV fluids among patients presenting with postoperative morbidity. The correlation of perioperative IV fluids and postoperative cumulative complications expressed by CCI was weak and meant that a small proportion of complications could be correlated to the amount of IV fluids. However, a critical threshold of 4400 ml IV fluid administration during the first 24 h was identified as an independent predictor of postoperative complication. More than 4400 ml IV fluids POD 0 was especially associated with increased respiratory complications, but neither pancreatic fistula nor delayed gastric emptying.

The issue on optimal fluid management in abdominal surgery is still open to debate. Previous studies assessing fluid therapy and outcome in PD perfectly reflects this situation. A fair amount of published studies including PD, predominantly retrospective cohort, described an increased rate of postoperative complications with increased fluids^9–13^. However, most randomized controlled studies^14–16^ failed to show any significant difference in postoperative outcome when comparing liberal to restrictive fluid therapy.

Comparison between studies on fluid therapy in PD is difficult because of the various definition of fluids (units, measure, and duration) and heterogeneous data. Moreover, most studies did not encompass the use of a systematic enhanced recovery protocol. Nevertheless, our center has a more restrictive fluid administration as in some other centers with a median intraoperative fluid administration of 3500 ml in comparison to 6000 ml for Kulemann et al.^10^ and 5000 ml for Weinberg et al.^9^. This is reflected in the intraoperative infusion rate too, as we have a median intraoperative infusion rate of 9 ml/kg/h (IQR 7–11), in comparison of 14 ml/kg/h for Eng et al., and corresponding to a restrictive fluid administration (< 10 ml/kg/h)^11,13^, although no standard definition exists. The rate of renal failure in the present study (3 and 5% in the < 4400 ml and ≥ 4400 ml groups, respectively) was in accordance to the 7% rate reported by Weinberg et al.^9^. Other parameters were comparable with the literature: similar demographics in term of age (mean age 65, going from 53.5 to 69.0 in a systematic review^17^), gender (male 52% versus 50.3%^10^ to 59%^9^), mean BMI (25.6 kg/m^2^ versus 25.8 kg/m^2^^12^ to 26 kg/m^2^^9^). As in most studies, a large majority of patients had malignant tumor as indication for PD (86% versus 73.4%^10^ to 88%^12^). The ASA score may be lower in the present study with 33.5% of patients with ASA-score of 3 or 4, which ranged from 34.5^10^ to 89.5%^12^ in other studies. In term of operative parameter, the mean operative time tend to be lower in our data (335 min versus 420 min^9^ to 445 min^11^) but with similar vascular resection rate (22% versus 24% for Behman et al.^12^) and blood loss (median blood loss 440 ml versus 350 ml^9^ to 909 ml^11^).

As avoiding fluid overload might improve postoperative outcome after PD, the way to achieve this euvolemia is still matter of debate. Goal directed fluid therapy requires cardiac output monitoring that may be invasive and lacks easily identifiable target. For these reasons it is a challenging measure to implement in daily clinical practice. Further guidance are awaited from the OPTIMISE II trial^18^, a multicenter international trial of cardiac output-guided fluid therapy with low-dose inotrope infusion compared with usual care in patients undergoing major elective gastrointestinal surgery. Meantime, a threshold of perioperative IV fluid is a simple and reproducible way to estimate the fluid balance. The ROC analysis identified a threshold of 4400 ml perioperative fluid for complications. Previously, specific thresholds for perioperative IV fluid were identified, for example for open colorectal surgery (> 3500 ml)^19^ or loop ileostomy closure (> 1700 ml)^20^. Although the above mentioned threshold of 3500 ml for perioperative fluids was determined for open colorectal surgery, it was previously used by extrapolation for pancreatodudenectomy^7^. Data for pancreatic resection are sparse and Bruns et al. identified a ratio (infusion rate/glomerula function rate) of 0.15 as predictive of pancreatic fistula^21^. Available data for liver surgery identified a threshold of an increased weight of 3.5 kg on the second postoperative day which was associated with increased major complications^22^, but no specific data on fluids were provided. In the present study, the weight difference did not vary between patients with or without complications, possibly indicating that fluid balance evaluation is a complex process.

Noteworthy, the 4400 ml threshold was related to increased complications and length of stay. Especially postoperative minor pulmonary complications were increased in patients receiving 4400 ml or more. Similarly, Eng et al. reported an increased rate of pulmonary complications (29 vs 5%, p < 0.01) related to intraoperative infusion when comparing patients receiving more or less than 13.95 ml/kg/h^11^. The meta-analysis of Garland et al^23^ found no difference between restrictive and liberal fluid therapy on overall morbidity (Odds Ratio 1.17, 95% CI 0.92–1.50) and pulmonary complications (Odds Ratio 0.69, 95% CI 0.43–1.10). While causes of postoperative respiratory complications are multifactorial, a recent systematic review^24^ identified enhanced recovery (risk ratio 0.35, 95% CI 0.21–0.58) and goal directed therapy (risk ratio 0.87, 95% CI 0.77 to 0.98) among other interventions as effective measure to reduce their occurrence. Postoperative pulmonary complications are probably also related to other complications, but the study design as well as the lack of date of occurrence of each complications prevented to conduct a separate detailed analysis of predictive factors, including other complications, of pulmonary complications. As enhanced recovery also encloses goal-directed fluid therapy, the direct effect of fluid management is difficult to isolate. The threshold of 4400 ml would be more on the restrictive side as opposed to the liberal side when considering the various cut-offs reported in the systematic review on fluid regimens in pancreatoduodenectomy by Garland et al^23^. A fear of a too restrictive fluid regimen is potential tissue hypoperfusion leading to an increased rate of renal failure or altered wound healing. For example, the RELIEF (Restrictive versus Liberal Fluid Therapy for Major Abdominal Surgery) trial observed an increased rate of acute kidney injury associated with restrictive fluid therapy (8.6% in the restrictive fluid group and 5.0% in the liberal fluid group) among high risk patient undergoing major abdominal surgery^25^. Of notice, in the RELIEF trial the median intravenous fluid during and up-to 24 h surgery was 3700 ml in the restrictive group and 6100 ml in the liberal group. In the present study no increased renal failure was observed in patients receiving less than 4400 ml.

Regarding pancreatic-specific complications such as delayed gastric emptying and pancreatic fistula, the present study did not observed increased risk associated with increased perioperative fluid infusion. This is in line with Garland et al.^23^ who reported in a meta-analysis no significant difference in occurrence of delayed gastric emptying or pancreatic fistula when comparing restrictive to liberal fluid therapy in PD patients. However, some retrospective studies not included in the abovementioned meta-analysis^26–28^ were suggesting an increase in pancreatic fistulas associated with excessive fluid. Moreover, a study from Bannone et al. reported an increased incidence of postoperative acute pancreatitis and pancreatic fistula in high-risk patients after pancreatoduodenectomy with an intraoperative fluid administration of ≤ 3 ml/kg/h^29^. In the present study, the median intraoperative fluid administration was 9 ml/kg/h and no increased rate of pancreatic fistula was observed between both groups.

Limitations of the present study are inherent to the retrospective analysis and to the limited study sample obtained in a single center. Moreover, enhanced recovery relies on compliance to protocol elements, including fluid management, which has altogether a strong influence on the postoperative outcome^7^. However, in order to avoid redundancy with previously published results^7^, this study focused on fluid management and impact of ERAS compliance was not assessed here. In addition, the amount of IV fluid administered in the first 24 h of surgery is not an entirely modifiable factor, as it is closely related to patient’s comorbidity, disease’s prognosis and surgery’s extent.

In order to validate and generalize this threshold of 4400 ml iv fluids on POD 0 and associated findings, further study including PD performed within an enhanced recovery setting need to be conducted. Once available, this will provide useful guidance on how to refine fluid management of patients undergoing PD to avoid both fluid overload and kidney insufficiency.

In conclusion, the present study emphasizes the potential deleterious consequence of fluid overload especially on pulmonary complications. A threshold for perioperative intravenous fluid at 4400 ml might be a useful indicator in the management after pancreatoduodenectomy within an enhanced recovery pathway.

## Methods

### Study design

This retrospective cohort study included all consecutive patients undergoing elective PD within an ERAS program in a tertiary referral centre (Lausanne University Hospital (CHUV), Switzerland) between October 2012 and June 2017. This cohort of patients was previously included and merged with data of three other institutions in a multicentre study^7^. Patients younger than 18 years old, and those who were opposed to the use of their data were not included. The study protocol was approved by the Institutional Review Board (CER-VD # 2016-01815) and patients gave informed consent. This study was reported in accordance with the STROBE guidelines^30^.

### Enhanced recovery protocol, fluid management and data collection

The enhanced recovery protocol for PD was initiated in October 2012 according to the ERAS guidelines^5^ and was previously detailed^7^. During the surgery, an infusion of Ringer Lactate at a rate of 3-5 ml/kg/h was initiated and bolus of 500 ml were given in case of decreased cardiac output (assessed by pulse pressure variation, oesophageal Doppler or transoesophageal echocardiography) or increased lactate with a minimal diuresis set at 0.5 ml/kg/h. Vasopressors were liberally used during and after the operation to counterbalance the vasodilator effect of epidural. In the postoperative period, an infusion of 1000 ml/24 h of Ringer Lactate was instilled, with 250 ml bolus of Physiogel in case of diuresis less than 2 ml/kg/4 h. Free oral drinks were encouraged from the day of surgery. Preoperative and demographic characteristics, intraoperative data and postoperative outcomes were prospectively collected using the ERAS Interactive Audit System (EIAS). The postoperative follow-up was 30 days.

### Fluids assessment and outcomes

Perioperative fluids were defined as intravenous (IV) infusion, including crystalloids, colloids and blood products given during the first 24 h of surgery (postoperative day 0: POD 0). The total IV volume administration on POD 0 (IV fluids POD 0) was used as parameter to define critical threshold.

Postoperative complications were graded using the Clavien classification^31^. Grade I and II were defined as minor and grade IIIa to IVb as major complications. Grade V was reported as mortality. The Comprehensive Complication Index^32^ (CCI) was calculated to obtain a continuous morbidity scale. Complications, including postoperative pulmonary complications were reported according to the EPCO (European Perioperative Clinical Outcome) definitions^33^. Accordingly, postoperative pulmonary complications included respiratory infection (antibiotics for suspected respiratory infection and with one or more criteria: sputum, lung opacities, fever, with blood cell > 12 × 10^9^/l), respiratory failure (postoperative PaO_2_ < 8 kPa, PaO_2_: FIO_2_ ratio < 40 kPa, or arterial saturation < 90% requiring oxygen therapy), pleural effusion (demonstrated by chest radiograph), atelectasis (lung opacification with shift toward affected area and compensatory over-inflation in the adjacent non-atelectatic lung), pneumothorax (air in the pleural space), bronchospasm (newly detected expiratory wheezing treated with bronchodilators) and aspiration pneumonitis (acute lung injury after the inhalation of regurgitated gastric content.

Primary outcome was overall postoperative morbidity. Secondary outcomes were major complications, CCI, and length of stay. Primary length of stay was the number of days spent in the hospital from the primary operation until discharge. Total length of stay was the addition of primary length of stay with the number of days following hospital readmission. Specific complications related to pancreas surgery were reported according to the respective ISGPS guidelines^34–37^.

### Statistical analysis

Descriptive statistics for categorical variables were reported as numbers and percentages, while continuous variables reported as median and interquartile ranges (IQR) or mean and standard deviations (SD). Categorical variables were compared with the Chi-Square or Fisher’s exact test. The Student’s t test and Mann–Whitney U test were used for continuous normally and non normally distributed data, respectively. Correlation between CCI and perioperative fluids was assessed by the Spearman’s test. The diagnostic ability was illustrated by a receiver operating characteristic (ROC) curve. An area under the curve (AUC) of > 0.7 was considered statistically significant. Threshold was identified according to the Youden index. Potential predictors of overall and major complications were assessed by binary logistic regression analysis and multiple logistic regression analysis was further performed including all previous independent variables with a *p* value < 0.1. All *p* values were two-sided and *p* < 0.05 was considered statistically significant. Data analysis was performed with SPSS version 25 (IBM Corp, Amonk, NY).
